# Adoption of large-scale medical equipment: the impact of competition in the German inpatient sector

**DOI:** 10.1007/s10198-021-01395-w

**Published:** 2021-11-08

**Authors:** Marie Dreger, Hauke Langhoff, Cornelia Henschke

**Affiliations:** 1 Department of Health Care Management, Technische Universität Berlin, Straße des 17. Juni 135, 10623 Berlin, Germany; 2 Berlin Centre for Health Economics Research (BerlinHECOR), Technische Universität Berlin, Straße des 17. Juni 135, 10623 Berlin, Germany; 3 Fakultät für Gesundheitswissenschaften Brandenburg, Brandenburgische Technische Universität Cottbus-Senftenberg, Cottbus, Germany

**Keywords:** Adoption of innovations, Imaging technologies, Hospital competition, Capacity planning, Panel data, Germany

## Abstract

The availability of large-scale medical equipment such as computed tomography (CT), magnet resonance imaging (MRI) and positron emission tomography (PET) scanners has increased rapidly worldwide over the last decades. Among OECD countries, Germany ranks high according to the number of imaging technologies and their applications per inhabitant. In contrast to other countries, there is no active governmental planning of large-scale medical equipment. We therefore investigated whether and how the adoption and distribution of CT, MRI and PET scanners in the German inpatient sector is subject to competition. Using a linear-probability model, we additionally examined the impact of regional, hospital- and population-based factors. In summary, our results indicate that the adoption rate by hospital sites decreases with the number of other sites being already equipped with the respective device and their proximity. However, the effect presumably depends on the technologies’ stage within the diffusion process. No influence regarding the amount of state subsidies could be identified. Furthermore, hospital size and university status strongly affect the adoption.

## Introduction

**Fig. 1:**
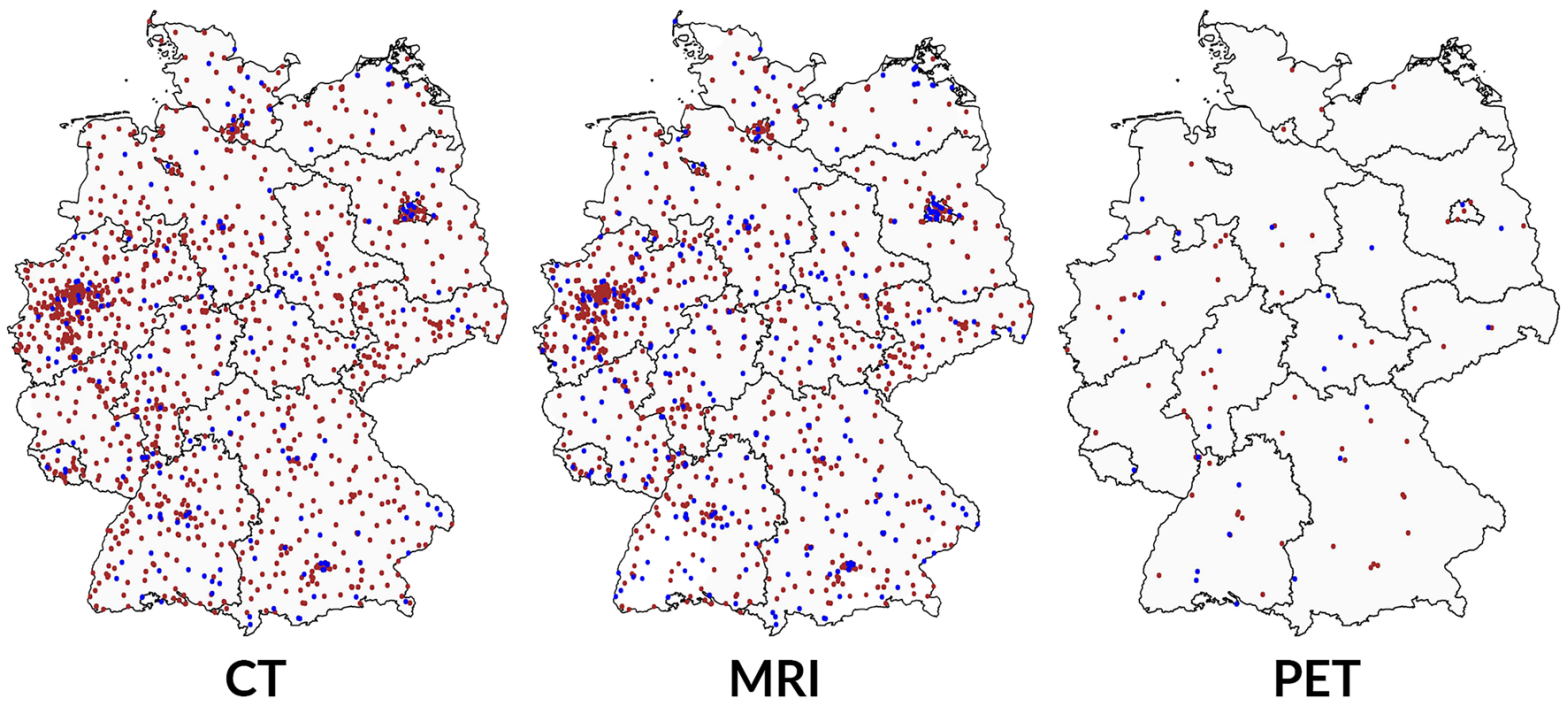
Distribution of CT, MRI and PET scanners in German hospital sites in 2017; blue mapped hospital sites represent new devices since 2010

The availability of large-scale medical equipment such as computed tomography (CT), magnetic resonance imaging (MRI) and positron emission tomography (PET) scanners alone or in combination with CT (PET-CT, hereafter: PET) has increased rapidly over the last decades. Regarding MRI, in 2020 or the latest available year, only Japan provides more units per population than Germany [1]. Regarding the number of conducted exams per capita, Germany even ranks first among the OECD countries [2]. The number of PET scanners quadrupled in the last 20 years [3], although Kotzerke et al. [4] complain that its use is restrained compared to other industrialised countries. In total, the number of the three scanners in the inpatient sector alone rose from 1058 in 1997 to 2688 in 2017 (+ 154 percent). The three imaging technologies vary in terms of their functions, application fields and purchase prices. CT creates slice images with the application of X-rays while MRI uses magnetic fields and radio waves avoiding ionising radiation [5]. In contrast, PET is a procedure in which positron emitters called radiotracer are brought into the tissue. By means of coincidence detection of the emitted gamma quanta, a very accurate image of the activity distribution in the body can be reconstructed [6]. Whether CT, MRI or PET is considered or has the better informational value depends on the body region, the tissue, and the question to be clarified. The main application field of CT is the imaging of bones and small calcifications, e.g. fractures or the presence of masses in lung cancer. MRI enables the diagnosis of pathologies in all parts of the body with the focus on soft-tissue contrast. By imaging metabolic processes, PET focuses on the detection of degenerated tissues, especially bone and metastatic tumours [5]. Figure 1 visualises the distribution of CT, MRI and PET scanners in German hospitals in 2017.

The widespread availability of imaging technologies may improve health outcomes by enabling diagnoses at an earlier stage of the disease or enhancing precision of diagnoses while also reducing the need for more invasive diagnostic tests [7–9]. However, the radiation exposure of CT and PET scans presents a harmful side effect. Some studies indicate that the rapid growth of performed CT scans and the amount of radiation exposure significantly result in an increased risk of cancer [10–13]. Additionally, incidental findings by imaging techniques may cause unfavourable consequences, e.g. the non-existence of treatment options for an observed condition, or the sole availability of invasive and hazardous follow-on interventions that finally may result in benign findings. Such situations may create anxiety for patients and affect their quality of life [14–16]. Overall, the clinical value of an increasing use of diagnostic imaging technologies remains unclear. This may partly be explained by conceptual and/or ethical challenges to conduct studies of high quality [9, 16, 17]. Furthermore, state-of-the-art imaging technologies are associated with substantial financial investments about more than 2 million euros per device with additional spending on necessary facility adaptations, operating and maintenance costs [18]. Together, these points raise concerns about the expanded diffusion of those technologies [7, 8, 19, 20].

To avoid an uncontrolled diffusion, several European countries such as Austria and France actively plan the number and/or the regional distribution of large-scale medical equipment [21, 22]. Similarly, most states in the USA have established so-called Certificate of Need (CON) programmes to facilitate coordinated planning of new services and facility constructions as well as the acquisition of large-scale medical equipment [16, 23]. In Germany, efforts of 1989 towards capacity planning of large-scale medical equipment were never fully implemented in practice and were finally repealed with the Second Statutory Health Insurance (SHI) Restructuring Act in 1997. Since then, investment and procurement decisions mostly have been the responsibility of the health care provider.

To better understand the context of our analysis, we briefly discuss special features of the German healthcare system with regard to investment and reimbursement measures for large-scale medical equipment. In inpatient sector, large-scale medical equipment as well as other capital expenditures have to be financed by the 16 federal states (*Bundesländer*). Hospitals being part of federal states’ hospital plans, which determine the number of specialties and bed capacities, are eligible for states’ funding of large-scale medical equipment. In contrast, investment funding from the federal states do not apply to the outpatient sector. University hospitals are entitled to state capital investment funding under the University Capital Investment Act. In sum, 98% of the hospital beds and therefore the majority of hospitals are part of investment funding [24]. Operating costs for large-scale medical equipment in the inpatient sector are usually covered by statutory and private health insurance funds and are reimbursed based on diagnosis-related groups (DRGs). Out-of-pocket payments may only occur if the imaging is done at patients' request. Although reimbursement differs in ambulatory care, patients receive CT, MRI and PET examinations free of charge from specialists licensed for SHI. However, for certain indications in ambulatory care the use of respective technologies is excluded from the benefit basket of SHI, especially with regard to PET [16].

Decisions on investments in large-scale medical equipment are at the discretion of respective state ministries and therefore depend on available budgetary restrictions and political priorities [24]. In the last decades, severe funding gaps have increased the use of equity capital and alternative financing models by hospitals [25]. Consequently, the federal states have gradually lost their influence as the main capital provider. A survey among 167 hospitals in 2015, and an analysis based on annual financial statements from a sample of 871 hospitals in 2013 estimate that about 50% of the investment costs are no longer covered by the states [26, 27].

In many respects, the German healthcare system is characterised by the coexistence of regulatory measures and competition [28]. Since 2004, competition among hospitals has been fostered with the introduction of a prospective hospital payment system based on DRGs [29]. This competitive approach towards self-regulation by market mechanisms, however, is counteracted by federal hospital planning and financing processes as described above. Hospitals try to amortise their investments in large-scale medical equipment through reimbursements of their operating costs by expanding their services and/or reducing personnel or other material costs [30].

Indeed, competition may lead to an optimal allocation of medical goods [31, 32] by shaping the providers’ behaviour [33]. Conversely, opponents claim that an unregulated market could lead to inefficiencies. Medical providers may be incentivised to increase their case numbers and overinvest in facilities and equipment [23, 32, 34]. Several studies examined the impact of competition among hospitals regarding the provision of health services [36–37]. However, only few studies have investigated the influence of competition on the adoption and diffusion of large-scale medical equipment. Following Baker and Atlas [38], managed care models inhibit the adoption of MRI units in the USA. Ladapo et al. [20], on the other hand, found that the adoption of a new CT scanner was less likely in competitive markets. Rye and Kimberly [39] conducted a literature search regarding the adoption of innovations by health care providers and observed both facilitated and impeded diffusion. The influence of competition on the adoption and diffusion of large-scale medical equipment has not been studied for the German context, although an uncontrolled proliferation was already assumed 20 years ago [40].

The main purpose of the study is to quantitatively assess predominant factors of adopting CT, MRI and PET scanners in German hospitals between 2010 and 2017. Thus, we focus on the research question whether and how the adoption of large-scale medical equipment is subject to competition in the absence of systematic capacity planning. Subsequently, this study aims to identify regional, hospital- and population-based determinants of distributional differences of large-scale medical equipment. In the following examination, the term adoption refers to the initial acquisition of a device by the hospital site.

## Methodology

### Data sources

The final study dataset comprises a broad range of sources. First, information on hospital characteristics was derived from the hospital structured quality reports according to § 136b Social Code Book Five (SGB V) from 2010 to 2017. Since 2012, hospitals have been obliged to publish those reports annually; prior to that, they were published biennially. Since no quality report was available for 2011, we interpolated the values of metric variables. All data was manually corrected after plausibility and consistency checks. Second, we remotely accessed the nationwide hospital discharge data (DRG statistics) via the Research Data Centre of the German Federal Statistical Office [41]. The DRG statistics captures anonymised information for all inpatient treatments. We further utilised the open access database of the German Federal Statistical Office for regional data. We determined the population density in the immediate vicinity of a hospital to an accuracy of 100 square metres using the Zensus 2011. Additionally, we studied the state hospital plans to obtain information about the type of financial support for large-scale medical equipment. Lastly, regional data on health services of outpatient care settings were provided by the Federal Association of SHI Physicians.

### Specification of variables

#### Dependent variable

We analysed the adoption of CT, MRI and PET scanners as representative large-scale medical equipment as they vary in their availability, are accompanied with the highest costs among imaging technologies and are most common across all countries and studies, thus, enabling international comparability [23, 31]. The outcome variables were dichotomous, with a value of 1 indicating if at least one device was available in a hospital site and 0 if otherwise.

#### Spatial competition

**Fig. 2:**
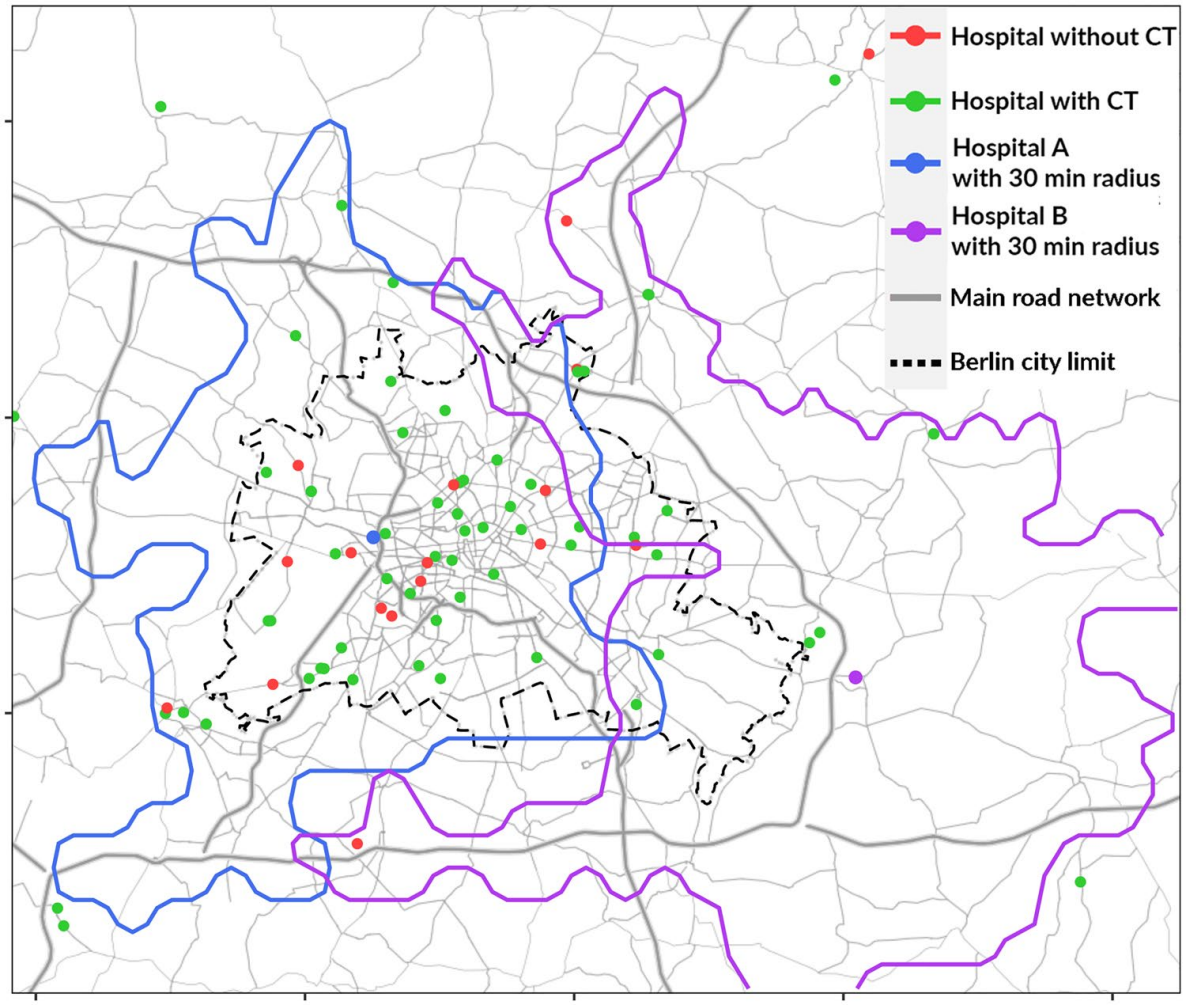
Hospital sites and travel time zones using the example of Berlin

Advanced medical equipment attracts physicians and patients to hospitals and is therefore seen as a competitive factor [9]. We hypothesise, that in the absence of regulatory restrictions, the adoption and diffusion of large-scale medical equipment follow competitive market mechanisms. Thus, we assume that a hospital site’s adoption depends on the degree of market concentration and the availability of CT, MRI and PET scanners in competing health service providers. According to the First Law of Geography by Tobler [42], “everything is related to everything else, but near things are more related than distant things”. Beyond this, Coenen et al. [43] state that competition among hospitals occurs in close proximity, delivered in nearby regions. Furthermore, two qualitative research studies identified competition as a direct determinant of the adoption of imaging technologies [9, 44]. We therefore tested these hypotheses within the German inpatient market. As a degree for market concentration we counted the number of hospital sites offering at least one of the respective devices in the surrounding (Fig. 2). Due to the binary coding and a lack of information on the market share of hospitals, we decided against the Herfindahl index. Adapting the Austrian planning specifications regarding accessibility [21], we modelled a radius around every hospital site of 30 (for CT), 45 (for MRI) and 60 min (for PET) by car.

#### Covariates

According to the studied literature, we identified the following variables as possible determinants. Due to administrative aggregation of some covariates, the data is captured at three different levels: hospital-specific, district and state level. Germany is divided into 401 districts organised within 16 states.

##### State level

Every German state has its own regulatory hospital planning procedures and may finance large-scale medical equipment according to three different models, or a combination thereof. As a result, Augurzky et al. [45] observe a state-specific regional variation of CT and MRI scanners provided in hospitals. First, in most states imaging technologies are financed according to individual agreements between the hospital and the state. Second, every state provides a lump-sum grant for hospital’s investment costs, mainly based on the number of beds. Third, three states have been switched to performance-oriented investment allowances which are calculated annually by the German DRG Institute since 2013. By incorporating the state-specific type and amount of funding we accounted for possible regulatory dependencies. We further included dummy variables to control for unobserved state-specific effects.

##### District level

Several studies indicate that the market size determines the adoption of medical technologies as it represents the level of demand [9, 31, 44]. Thus, larger cities are associated with a higher rate of adoption [39, 46]. We therefore accounted for the population density of the districts in Germany. In addition, the need for and the use of health services depends on patients’ morbidity [35]. In the case of imaging technologies, furthermore, we assumed repeated use per patient due to chronic diseases such as cancer [4, 16]. In this way, we operationalised a variable $M$ that accounts for device-related morbidity per district. We calculated a patient-specific likelihood of receiving a CT, MRI or PET examination based on age, sex and diagnoses. This value was aggregated for all inpatient cases in one district provided by the DRG Statistics database (Formula 1, for MRI and PET analogously) [41].

1.1 $$M_{{{\text{district}},{\text{CT}}}} = \sum\limits_{{i = 1}}^{n} {P_{i} } = \sum\limits_{{i = 1}}^{n} P ({\text{patient}}_{i} {\text{ receives CT scan }}|{\mkern 1mu} {\text{age}}_{i} {\text{, sex}}_{i} {\text{, discharge diagnosis}}_{i} ),$$

*n*—number of patients in the district.

The individual probability ${P}_{i}$ is estimated using the frequencies of the nationwide cases:

1.2 $$P({\text{patient}}_{i} {\text{ receives CT scan }}|{\mkern 1mu} {\text{age}}_{i} {\text{, sex}}_{i} {\text{, discharge diagnosis}}_{i} ) = \frac{{N_{{{\text{CT}}}} {\text{(age}}_{i} {\text{, sex}}_{i} {\text{, discharge diagnosis}}_{i} )}}{{N_{{{\text{total}}}} {\text{(age}}_{i} {\text{, sex}}_{i} {\text{, discharge diagnosis}}_{i} )}}.$$

In Germany, many ambulatory devices and thus services are provided outside the hospital [47]. Therefore, we included the number of SHI-affiliated physicians of radiology as an approximation of outpatient provided CT and MRI scanners. Similarly, we considered the sum of SHI-affiliated physicians of radiology and nuclear medicine regarding PET scanners.

##### Hospital-specific level

According to the population density on district level, we incorporated a hospital-specific variable which depicts the degree of urbanity. This is modelled by the population density within a radius of one kilometre around the hospital. Both, Sandoval et al. [9] and Abedini et al. [44] categorise hospital characteristics as an important factor influencing the adoption of large-scale medical equipment. An economic operation of large-scale medical equipment requires a high utilisation rate for skimming the economies of scale [16, 48]. Hence, the number of beds as an approximation for organisational size represents a determining factor. Furthermore, hospital size is accompanied by larger personnel and more budgetary resources that facilitate the access to alternative financing models [9, 49]. From this point of view as well, the type of ownership was considered as an important determinant, as it implies different corporate strategies and utilisation patterns of equity capital. Accordingly, in 2013 only 7% of private hospitals were unable to make investments, while the proportion of public and not-for-profit institutions were 62 and 40%, respectively [50]. Since we assume that the type of ownership affects how responsive a hospital is to competition, we additionally included the interaction of these variables in our analysis. Finally, the university status entitles hospitals to receive extra funding for their research and teaching portfolios. Thus, university hospitals are considered as early adopters of new technologies [51, 52]. Table 1 summarises all included variables as well as their sources and descriptive statistics.

**Table 1:** Descriptive statistics

Level	Label	Definition	Type of variable	Data period	Data source	Min	Mean	Max
Dependent variable								
Hospital sites	Availability of CT scanner	Availability of the respective device in the hospital site under investigation	Binary (0 = ‘no’, 1 = ‘yes’)	2010–2017^1^	Structured quality reports according to § 136b SGB V	1321 (2010)^2^		1583 (2017)^2^
	Availability of MRI scanner					970 (2010)^2^		1279 (2017)^2^
	Availability of PET scanner					69 (2010)^2^		95 (2017)^2^
Independent variables								
Ego-centred	No. of hospital sites with CT in 30 min	Number of hospital sites with the respective device within a certain distance measured by driving minutes	Metric	2010–2017^1^	Structured quality reports according to § 136b SGB V	0	13.44	102
	No. of hospital sites with MRI in 45 min					0	11.36	89
	No. of hospital sites with PET in 60 min					0	2.77	12
State level	Amount of financial support	Total funding per bed in euro	Metric	2010–2017	Working Group of the State Health Authorities^3^, Federal Statistical Office	2439.48	5498.00	11,289.52
	Type of support	Support for large-scale equipment: 1 = ‘individual agreements’/2 = ‘individual agreements + lump-sum grant’/3 = ‘performance-oriented investment allowances’	Nominal	2010–2017	State hospital plans			
District level	Population density	Number of inhabitants per square kilometre	Metric	2010–2017	Federal Statistical Office	36.13	523.11	4712.76
	Morbidity CT	Population prevalence of imaging relevant diseases	Metric	2010–2017	Research Data Centre of the German Federal Statistical Office	1338.19	1815.17	2250.96
	Morbidity MRI					520.71	691.69	886.00
	Morbidity PET					10.30	16.45	26.84
	Practitioners of radiology	Number of practitioners of radiology	Metric	2010–2017	Federal Association of SHI Physicians	0.00	N/A^4^	180.50
	Practitioners of radiology and nuclear medicine	Number of practitioners of radiology and nuclear medicine	Metric	2010–2017	Federal Association of SHI Physicians	0.00	N/A^4^	229.25
Hospital-specific level	No. of beds	Number of beds	Metric	2010–2017^1^	Structured quality reports according to § 136b SGB V	0.00	249.23	3,213
	Urbanicity	Population density within a radius of one kilometre around the hospital	Metric	2011	Zensus 2011	0	60,222.88	452,704.00
	University status	University hospital according to § 5 para. 1 no. 1 KHG	Binary (0 = “no”, 1 = “yes”)	2010–2017^1^	Structured quality reports according to § 136b SGB V	43 (2010)^2^		49 (2017)^2^
	Type of ownership	Type of hospital’s ownership: 1 = ‘not-for-profit’/2 = ‘private (for-profit)’/3 = ‘public’	Nominal	2010–2017^1^	Structured quality reports according to § 136b SGB V			

*KHG* Hospital Financing Act (*Krankenhausfinanzierungsgesetz*), *No.* Number

^1^Values for 2011 were interpolated

^2^Number of items with the expression ‘yes’ (year),

^3^Arbeitsgemeinschaft der Obersten Landesgesundheitsbehörden (AOLG)

^4^No averaging possible due to grouped data

### Statistical analysis

We addressed our research questions by applying a linear-probability model (LPM). An LPM makes use of a linear regression in order to explain qualitative events. Using a binary-coded dependent variable the coefficients of a linear model can be interpreted as the change in the probability of a defined event given a one-unit change in the independent variable, holding all other co-variables constant [53]. The core advantages of LPM compared to non-linear models for limited dependent variables (e.g. logit or probit estimators) are the way of estimation and an intuitive interpretation of the results. Additionally, it may avoid complex procedures for comparing effect sizes across models which occur in terms of non-linear models [54]. However, due to the binary result of the dependent variable, the assumption of homoskedasticity of the model errors is necessarily violated. To ensure the validity of the statistical tests we therefore calculate heteroscedasticity-robust standard errors [55]. Some combinations of independent variables’ values can provide predicted probabilities that are less than zero or greater than one. Hence, an interpretation of the results needs to include plausibility checks.

There are several yearly observations for every hospital site in our data: between 1 and 8 (6 on average). To account for this panel data structure, we computed a random effect on the level of the hospital site [56]. This specification is analogous to a two-level multilevel model where the yearly observations are defined as level one and the hospital sites are defined as level two [57]. The combination of the linear-probability model with the computation of a random error fit our dependent variable as well as the structure of our data. The specification of random effects was preferred to fixed effects as there was not enough development over time and thus no variance to be analysed. Logarithmic transformation was appropriate for the number of SHI-affiliated physicians, hospital beds and population density to fit into linear model estimation. Accordingly, the applied model can be described as follows:

2 $$\begin{array}{*{20}c} {\hat{P}\left( {y_{{ij}} = 1} \right) = \left( {\beta _{{0~}} + ~\zeta _{{0j}} } \right) + ~\beta _{1} x_{{1ij}} + \ldots + \beta _{n} x_{{nij}} + ~\varepsilon _{{ij}} } \\ {{\text{for observation}}~i{\text{ in the clinic site }}j} \\ \end{array} ,$$

where $\widehat{P}$ is the probability to have an event and ${\zeta }_{0j}$ represents the random intercept. In the results, the random effect is expressed by its standard deviation and describes the distribution of the model error component that can be attributed to the belonging of a particular clinic site. ${\varepsilon }_{ij}$ is the remaining model residual on level one and is described by its standard deviation. The relation between these two components represents the relative importance of both levels for the outcome. The explanatory power of the model can be described by the reduction of the overall error variance compared to that of a zero model. The performance of the full model was tested by means of a Wald test. As we apply the ego-centred geographical regional aggregation of our main independent variables, we took into account a partial reduction of their variance and their explanatory power [58]. However, all variables showed an acceptable amount of distribution. Using correlation analyses according to Pearson, bivariate relationships could be mapped. Finally, we computed successive models where we varied the distance from 10 to 90 driving minutes at which the availability of the respective devices in the local area were measured. Thus, a main advantage of ego-centred operationalisation may be realised. By comparing the ego-centric neighbourhoods with varying distances, the spatial reference of the acting mechanisms could be determined. This is important, because the results of such analyses depend on the definition of the spatial units under investigation [58, 59].

## Results

The following section presents the observed predominant factors enhancing the adoption of CT, MRI and PET scanners in German hospital sites considering the years between 2010 and 2017 (Table 2).

**Table 2:** LPM regression coefficients and standard errors for CT, MRI and PET

	CT		MRI		PET	
	Coefficient	Standard error	Coefficient	Standard error	Coefficient	Standard error
Ego-centred						
No. of hospital sites with CT in 30 min	− 0.001*	(0.001)				
No. of hospital sites with MRI in 45 min			− 0.002***	(0.001)		
No. of hospital sites with PET in 60 min					− 0.004***	(0.001)
State level						
Amount of financial support	0.002	(0.002)	− 0.003	(0.003)	− 0.002	(0.001)
Type of support = 1	Ref	(.)	Ref	(.)	Ref	(.)
Type of support = 2	0.207**	(0.068)	− 0.018	(0.075)	− 0.040	(0.028)
Type of support = 3	− 0.005	(0.010)	0.011	(0.011)	− 0.001	(0.003)
District level						
Population density (ln)	− 0.023**	(0.008)	0.027**	(0.009)	0.012***	(0.003)
Morbidity CT	0.003	(0.004)				
Morbidity MRI			− 1.232	(1.074)		
Morbidity PET					− 0.000	(0.000)
Practitioners of radiology (ln)	0.006	(0.006)	0.008	(0.008)		
Practitioners of radiology and nuclear medicine (ln)					0.009***	(0.002)
Hospital-specific level						
No. of beds (ln)	0.110***	(0.004)	0.112***	(0.005)	0.016***	(0.001)
Ownership: not-for-profit	Ref	(.)	Ref	(.)	Ref	(.)
Ownership: private (for-profit)	− 0.026	(0.020)	− 0.020	(0.022)	0.008	(0.007)
Ownership: public	0.002	(0.017)	0.025	(0.019)	0.003	(0.006)
Not-for-profit * No. of hospital sites with the device	Ref	(.)	Ref	(.)	Ref	(.)
Private (for-profit) * No. of hospital sites with the device	− 0.001	(0.001)	0.000	(0.001)	− 0.002	(0.002)
Public * No. of hospital sites with the device	0.001	(0.001)	0.004***	(0.001)	0.002	(0.001)
No university status	Ref	(.)	Ref	(.)	Ref	(.)
University status	0.045	(0.043)	0.114*	(0.048)	0.435***	(0.017)
Urbanicity (ln)	0.048***	(0.006)	0.047***	(0.007)	0.003	(0.003)
Time						
Year of acquisition = 2010	0.000	(.)	0.000	(.)	0.000	(.)
Year of acquisition = 2011	− 0.001	(0.006)	0.004	(0.007)	0.004*	(0.002)
Year of acquisition = 2012	0.049***	(0.007)	0.083***	(0.009)	0.005**	(0.002)
Year of acquisition = 2013	0.050***	(0.009)	0.096***	(0.011)	0.007***	(0.002)
Year of acquisition = 2014	0.044***	(0.011)	0.103***	(0.013)	0.007***	(0.002)
Year of acquisition = 2015	0.035**	(0.012)	0.102***	(0.014)	0.008***	(0.002)
Year of acquisition = 2016	0.042**	(0.014)	0.121***	(0.017)	0.013***	(0.002)
Year of acquisition = 2017	0.047**	(0.016)	0.133***	(0.018)	0.016***	(0.002)
Constant	− 0.364***	(0.100)	− 0.646***	(0.109)	− 0.153***	(0.029)
sd (constant)	0.362***	(0.005)	0.393***	(0.006)	0.149***	(0.002)
sd (residual)	0.146***	(0.001)	0.180***	(0.001)	0.051***	(0.000)
Observations	16,580		16,580		16,580	

Variables in the model, not listed in the table: federal state

*ln* natural logarithm, *No.* number, *Ref.* reference category

*p < 0.05, **p < 0.01, ***p < 0.001

**Fig. 3:**
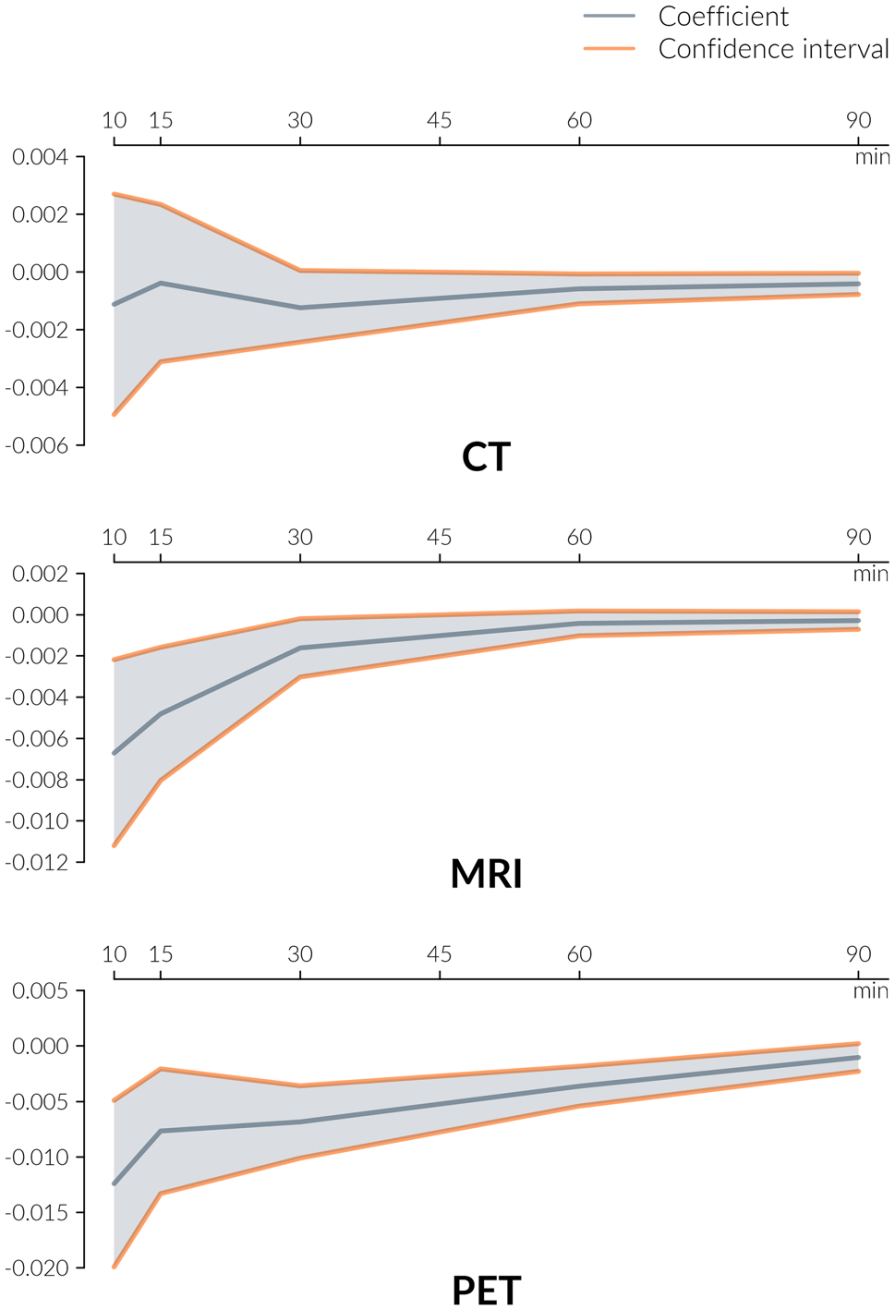
Ego-centred geographical regional aggregation for a radius from 10 to 90 min

The LPM revealed that the degree of market concentration was statistically significant for all devices (CT: − 0.001, *p* < 0.05; MRI: − 0.002, *p* < 0.001; PET: − 0.004, *p* < 0.001). Thus, a hospital site was 0.1, 0.2 and 0.4 percent less likely to offer a CT, MRI and PET scanner by every other site being already equipped in the surrounding polygon based on a distance of 30, 45 and 60 driving minutes, respectively. As the value of the explanatory variable reaches up to 102 CT, 89 MRI and 12 PET scanners, the model can explain up to 10, 17.8 and 4.8% of the overall probability. The results of the successive models based on the varying distances are shown in Fig. 3. It additionally demonstrates that the observed influence declines exponentially by the examined distance. With increasing distance, the effect converges towards zero. Regarding PET scanners, for instance, the effect increased by 3.4 times by every available relevant device in 10 driving minutes. However, for CT scanners, no clear tendency can be recognised. According to the confidence intervals, effects can be potentially identical for all distance values. This observation is consistent with the relatively weak effect observed for CT scanners in the LPM.

### State level

According to our hypothesis, no evidence was found regarding the amount of federal investment. However, the distribution of state funding via lump-sum in addition to individual agreements between state and hospital increased the hospital’s adoption of CT by 20.7 percent (*p* < 0.01).

### District level

**Fig. 4:**
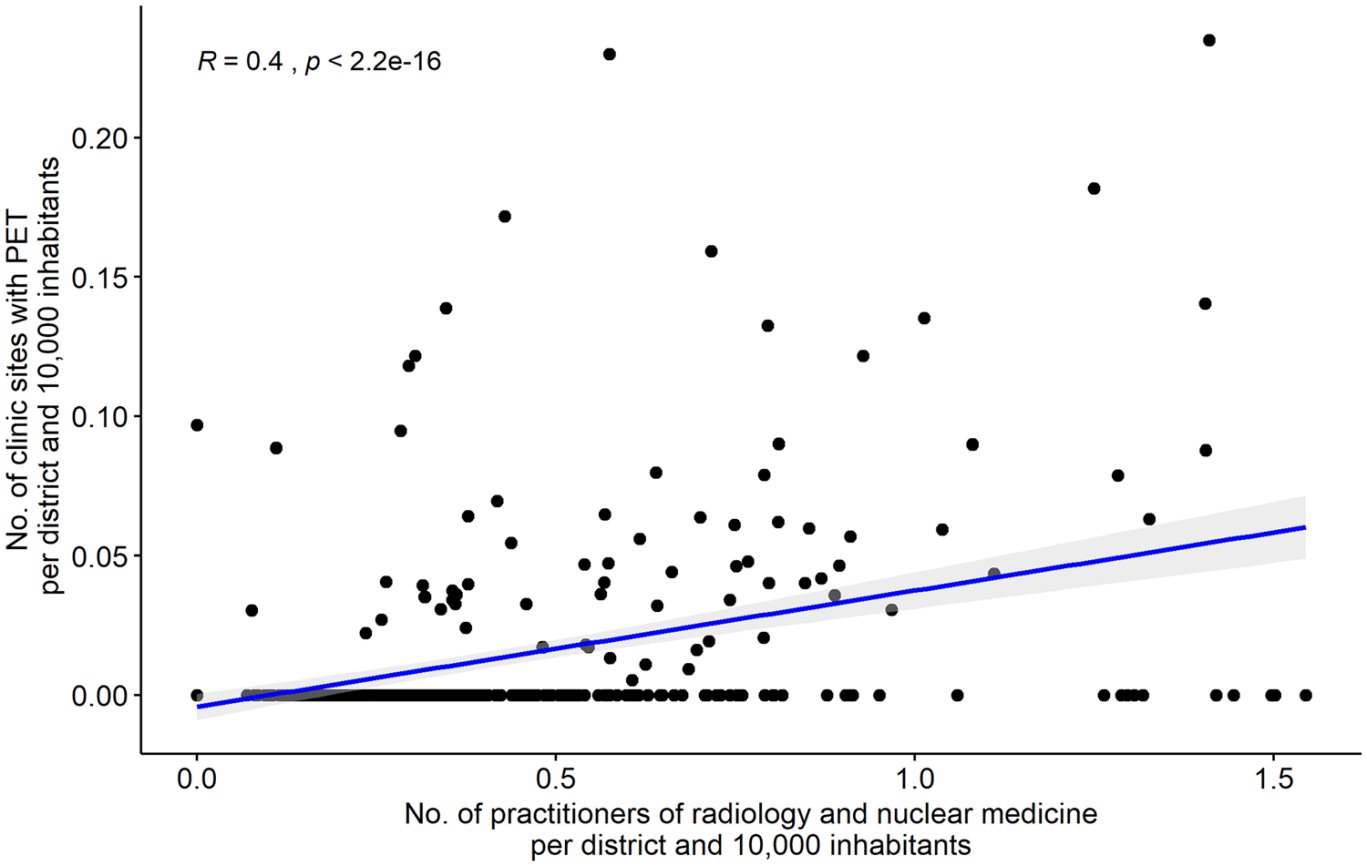
Pearson correlation for the number of outpatient and inpatient PET

At district level, we observed a positive significant effect of SHI-affiliated physicians of radiology and nuclear medicine on the adoption of PET scanners (0.009, *p* < 0.001). This is in line with the Pearson correlation coefficient (*R* = 0.4, *p* < 0.001). Thus, the approximate number of outpatient and inpatient PET scanners per inhabitant and district indicate a positive linear correlation (Fig. 4). Furthermore, evidence was revealed for the population density on district level. While a high population density increases the probability for the hospital site’s availability of MRI (0.026, *p* < 0.01) and PET (0.012, *p* < 0.01), the effect is reversed for CT scanners (− 0.023, *p* < 0.01). No influence could be found for the device-specific morbidity.

### Hospital-specific level

At hospital-specific level, we observed strong positive evidence of the logarithmic number of beds for all devices (CT: 0.112, *p* < 0.001; MRI: 0.110, *p* < 0.001; PET: 0.016, *p* < 0.001). Furthermore, we identified significant differences regarding the university status for MRI and PET scanners. Thus, university hospitals were 1.1 and 4.4 percent more likely to provide an MRI and PET scanner, respectively. We further observed positive significant correlations between the local degree of urbanity and the adoption of CT (0.048, *p* < 0.001) and MRI scanners (0.047, *p* < 0.001). The interaction between the ownership type and competition was significant only for MRI scanners. Public hospitals thus seem to be more responsive to competition than not-for-profit hospitals.

### Overall results

The time-specific variables reflect the increase of the devices over the years. Regarding MRI and PET scanners, the probability increased by time, whereas the adoption of CT scanners was more prominent in 2012 and 2013. The random effect is expressed in form of its standard deviation. It describes the distribution of the model error component that can be attributed to a particular hospital site. As the random effect is larger than the remaining model error, the most unobserved explanatory factors for having or not having a device are characteristics of the clinic site or factors that are strongly correlated with them, for example local characteristics.

## Discussion

The study aimed to determine the predominant factors concerning the adoption of CT, MRI and PET scanners in the German hospital sector. Thus, we primarily analysed, whether the distribution of these imaging technologies follows competitive rules in absence of regulatory planning. Indeed, our results indicate that the amount of federal financing is not determining the adoption of CT, MRI and PET scanners. Additionally, we found strong evidence that the adoption of large-scale medical equipment is negatively influenced by available CT, MRI and PET scanners at competing health care providers in the regional surrounding. This is consistent with Ladapo et al. [20] who found that American hospitals were more likely to adopt new imaging technologies in less competitive markets. Competition affects the investment decision, as equipment is an essential component of structural and process quality [60]. The DRG system promotes competition between hospitals, which strive to improve quality and efficiency for attracting patients and physicians [61]. Research shows that advanced technology such as large-scale medical equipment attracts personnel and increases referrals by physicians and patients themselves [9, 63–64]. Our analysis confirms competition as a direct determinant of adopting large-scale medical equipment and is thus in line with the conceptual framework by Sandoval et al. [9]. Furthermore, our results correspond to the first law of geography [42] since this influence declines with growing distance. In accordance with Coenen et al. [43], we thus confirm that hospitals compete predominantly in the nearby region. The strongest evidence could be observed regarding PET scanners even though the cut-off value of 60 min was twice as high compared to CT scanners. This may be explained by (1) the lower overall distribution and (2) a patient care that is more specialised for PET compared to CT scanners being part of basic emergency care [65]. This correponds with the Austrian planning values according to which CT scanners should be accessible in 30 and PET scanners in 60 min [21]. Thus, less widespread availability is required for PET scanners. In the long term, the latter aspect might be of increasing importance with regard to the diffusion of CT scanners due to the reform of emergency services in Germany, which has been in the process of implementation since 2018. Accordingly, hospitals receive financial remuneration supplements if they participate in emergency care and are able to demonstrate the structural requirements of 24-h availability of CT [66]. However, the effect for CT scanners converges towards zero by the examined distance and is barely existent for 30 min. It can thus be assumed that the inpatient sector has reached a high saturation of CT scanners. This could be supported by the cartographic visualisation of hospital sites. In addition, the stronger effect for PET in comparison to CT and MRI scanners may be due to higher purchase prices and so the decisions are more likely to be based on economic reasons. Furthermore, indication restrictions in the outpatient setting may influence the results obtained. Accordingly, the hospital sites represent the main service provider of PET and thus competition between them has a higher influence.

On district level, PET scanners in outpatient settings influenced the adoption in the examined hospital sites. The observed positive correlation contradicts the assumption of the health sectors acting as cooperative substitutes [67]. This may be attributed to the hospital acting as an operator of a medical treatment centre. According to the Federal Association of SHI Physicians, in 2018 almost every second medical treatment centre was run by a hospital operator [68]. Legally, they belong to outpatient sector; nevertheless, some hospital operators do not differentiate for their equipment in the quality reports. Therefore, data-inherent bias in form of double counts should be taken into consideration. Interestingly, the analysis revealed population density as negative determinant of adopting CT scanners. In contrast, many studies state that market size represented by the number of potential patients determines the adoption of technologies [9, 20, 39, 44, 46].

Regarding the level of the hospital site, as anticipated, the number of beds was positively correlated with the adoption of large-scale medical equipment. This substantiates previous findings by Ladapo et al. [20] as well as the qualitatively derived adoption models for imaging technologies of Sandoval et al. [9] and Abedini et al. [44]. This is plausible, since expensive diagnostic equipment first requires high investments and then a large number of patient examinations to recoup the expenditures. This in turn may be associated with a high number of beds [9, 16, 48, 49]. Contrary to our expectations, we did not observe any effect concerning the type of ownership. As hypothesised, the university status was positively correlated with the adoption of MRI and PET scanners while no evidence could be provided regarding CT scanners. This observation confirms the assumption of a saturated market and categorisation of CT into an advanced stage of diffusion [69], as university hospitals are considered as first adopters of new technologies [51, 52]. The technology of CT has been introduced to the German healthcare system in the 1970s, followed by MRI scanners a decade later, while PET scanners have been first available in 1988 [70]. Altogether, PET scanners may be classified in an earlier, MRI in a middle and CT in a late adoption and diffusion stage. Consequently, PET scanners revealed the highest influence regarding early adopters.

Lastly, the linear-probability model revealed that the years 2012 and 2013 were associated with a higher adoption rate of CT scanners than the other years. According to Ex and Henschke [71], changes regarding the reimbursement substantially incentivises the technology’s utilisation. Nevertheless, between 2010 and 2017, no relevant changes regarding the reimbursement of CT examinations could be identified. Since no progressive effect over time could be observed, this substantiates the hypothesis of a CT saturated inpatient market. On the other hand, the effect for MRI and PET scanners increased by time, hence, indicating an earlier stage of diffusion [69].

To be noted, a special feature regarding large-scale medical equipment is the long-term commitment. Therefore, increasing competition among providers may inhibit the adoption rate in the nearby region but fails at rationalising existing structures. This is also due to federal countermeasures in German inpatient sector, for example, regarding the survival of non-economic hospitals [72], which in fact also have other reasons such as access to inpatient care faculties, for instance. Presumably, neither clean competitive nor effective federal controlled diffusion of large-scale medical equipment occurred in the past in Germany. As a result, especially regarding CT scanners, which were implemented about two decades earlier than PET scanners, a highly saturated market could be observed. Nowadays, our results indicate that hospital sites adopt large-scale medical equipment in a rational behaviour. Finally, the comparison between CT, MRI and PET scanners leads to the conclusion that the competitive impact on the adoption of large-scale medical equipment depends on the technologies’ diffusion stage and is therefore stronger in terms of PET and MRI scanners. In a long-term perspective, the distribution spread to rural areas, thus, providing more equity regarding the density of devices. In the short term, however, an unwarranted variation [73] may result as the adoption of new devices first occurs in urban regions and university hospitals. Consequently, an undersupply in rural, economically unattractive regions can be observed. This problem may be solved by governmental regulation which directs the distribution of large-scale medical equipment even in economically unprofitable regions to ensure nationwide supply. However, this requires extensive financial resources and should be part of a holistic reorganisation of the German hospital sector.

The combination of competition and governmental countermeasures seems to lead to overcapacities possibly incentivising supply-induced demand [13, 74, 75]. Unfavourable consequences not only include inefficient use of resources in the German healthcare system but also put patients at risk of avoidable radiation exposure and incidental findings. In order to reduce these risks, health policy makers can promote a rational use of CT, MRI and PET examinations or restrict the number of devices. While the latter one might be difficult to realise in practice as no general guideline concerning the ideal number of scanners per million population exists [2], promoting measures incentivising the rational use of imaging procedures should be further strengthened. Those include clinical guidelines but also campaigns such as Choosing Wisely led by medical societies that publish recommendations on cases when imaging procedures are not necessary [76, 77]. Financial (dis)incentives may strengthen possible effects. Berger and Czypionka [75], for example, found reduced rates of MRI examinations in Austria when SHI funds restrict the access by setting requirements for referrals.

## Limitations

It is plausible that the following limitations may have influenced the results obtained. Due to several data sources and therefore administrative aggregation, the data is captured at three different levels: hospital-specific, district and state level. As we conducted the analysis on hospital sites’ individual level, the ecological inference may be fallacious [78]. Furthermore, the administrative boundaries may not represent the hospital sites catchment area. We were able to eliminate the modifiable areal unit problem (MAUP) [58] solely for the market concentration. Due to the binary outcome variable, we could not capture the number of CT, MRI and PET scanners within one hospital site and, beyond that, account for new acquisitions in case of replacement of old devices. Furthermore, it was not possible to control for the specialisation of a hospital site as well as for effects at the individual level of the decision maker. As the analysis revealed, the strongest unobserved determinants are inherent in the clinic site or factors that are strongly correlated with it. Possible determinants affecting the adoption across hospital sites, we were not able to control for, are changes in clinical guidelines, launches of new devices, novel financial assistance models and marketing methods of the vendor [44].

## Conclusion

In summary, our results indicate that competition among hospitals influences the adoption of large-scale medical equipment in absence of regulatory planning. The adoption rate decreases with the number of competing providers and their proximity. The comparison between CT, MRI and PET scanners suggests the more obsolete a technology, the lower the effect. Depending on the diffusion stage of the technology, determinants of adopting large-scale medical equipment differ. On hospital-specific level, the number of beds predominantly determines the adoption of CT, MRI and PET scanners. In addition, regarding MRI and PET scanners, the university status and a high population density on district level affect the adoption. With respect to CT scanners, the adoption mainly occurs in sparsely populated regions but no progressive effect over time could be revealed. This substantiates the categorisation of CT scanners into an advanced stage of diffusion, where devices primarily spread to rural areas, thus providing more equity in access to care. At first glance, competition seems to benefit the provision of large-scale medical equipment, however, fails at rationalising the grown structures. Consequently, the number of CT, MRI and PET scanners continuously rises even though the adoption rate may be inhibited. The resulting partial oversupply not only increases healthcare expenses but can also lead to overuse by service providers and thus harm patients. Finally, the coexistence of competition and governmental countermeasures in the German hospital sector restrains the effectiveness of either system. These observations raise concerns whether the German healthcare system requires an active planning programme of large-scale medical equipment or whether to give free rein to the invisible hand of self-regulating market mechanisms. This is an important issue in the context of a reorganisation of the German inpatient sector.

## Data Availability

Partly available.

## Code availability

Custom code.
